# Dominican amber net-winged beetles suggest stable paleoenvironment as a driver for conserved morphology in a paedomorphic lineage

**DOI:** 10.1038/s41598-022-09867-6

**Published:** 2022-04-06

**Authors:** Vinicius S. Ferreira, Alexey Solodovnikov, Michael A. Ivie, Robin Kundrata

**Affiliations:** 1 Natural History Museum of Denmark, Zoological Museum, University of Copenhagen, Universitetsparken 15, 2100 Copenhagen, Denmark; 2 Montana Entomology Collection, Marsh Labs, Montana State University, Room 5, 1911 W. Lincoln Street, Bozeman, MT 59717 USA; 3 Department of Zoology, Faculty of Science, Palacky University, 17. listopadu 50, 77146 Olomouc, Czech Republic

**Keywords:** Palaeontology, Taxonomy, Evolution, Zoology, Entomology

## Abstract

Paedomorphosis is a heterochronic syndrome in which adult individuals display features of their immature forms. In beetles, this phenomenon occurs widely in the superfamily Elateroidea, including the net-winged beetles (Lycidae), and, due to the usual flightlessness of paedomorphic females, it is hypothesized to cause speciation rates higher than in non-paedomorphic lineages. However, some fossils of paedomorphic lycids do not support this with palaeobiological data. Discovery of new Lycidae fossils attributed to the West Indian extant paedomorphic genus *Cessator* Kazantsev in the Dominican amber also suggests morphological stasis within this genus in the Greater Antilles. We describe *Cessator anachronicus* Ferreira and Ivie, sp. nov. based on adult males, as well as the first ever recorded fossil net-winged beetle larva of the same genus. We propose that the relatively young age of the studied fossils combined with the stable conditions in the forest floor of the Greater Antilles through the last tens of million years could explain the exceptionally conserved morphology in the net-winged beetles affected by the paedomorphic syndrome.

## Introduction

Paedomorphosis is a heterochronic syndrome in which adult individuals display features of their immature forms^1,2^. In beetles (Coleoptera), the paedomorphic syndrome can be expressed as a mosaic of characteristics, which can include the reduction of sclerotization and/or loss of flight ability, miniaturization of morphological structures, the predominance of a K-reproductive strategy and enhanced fecundity of females^3–7^. In extreme cases, adult paedomorphic beetles can be completely larviform and fully resemble their larval stages^6,8^. Despite the conspicuousness of this syndrome in beetles, especially in the superfamily Elateroidea, which includes click-beetles (Elateridae), fireflies (Lampyridae) and rail-road worms (Phengodidae)^3,4,6,7,9–14^, the processes that generate these modifications and overall effects of paedomorphosis remain largely unknown and poorly studied.

**Figure 1:**
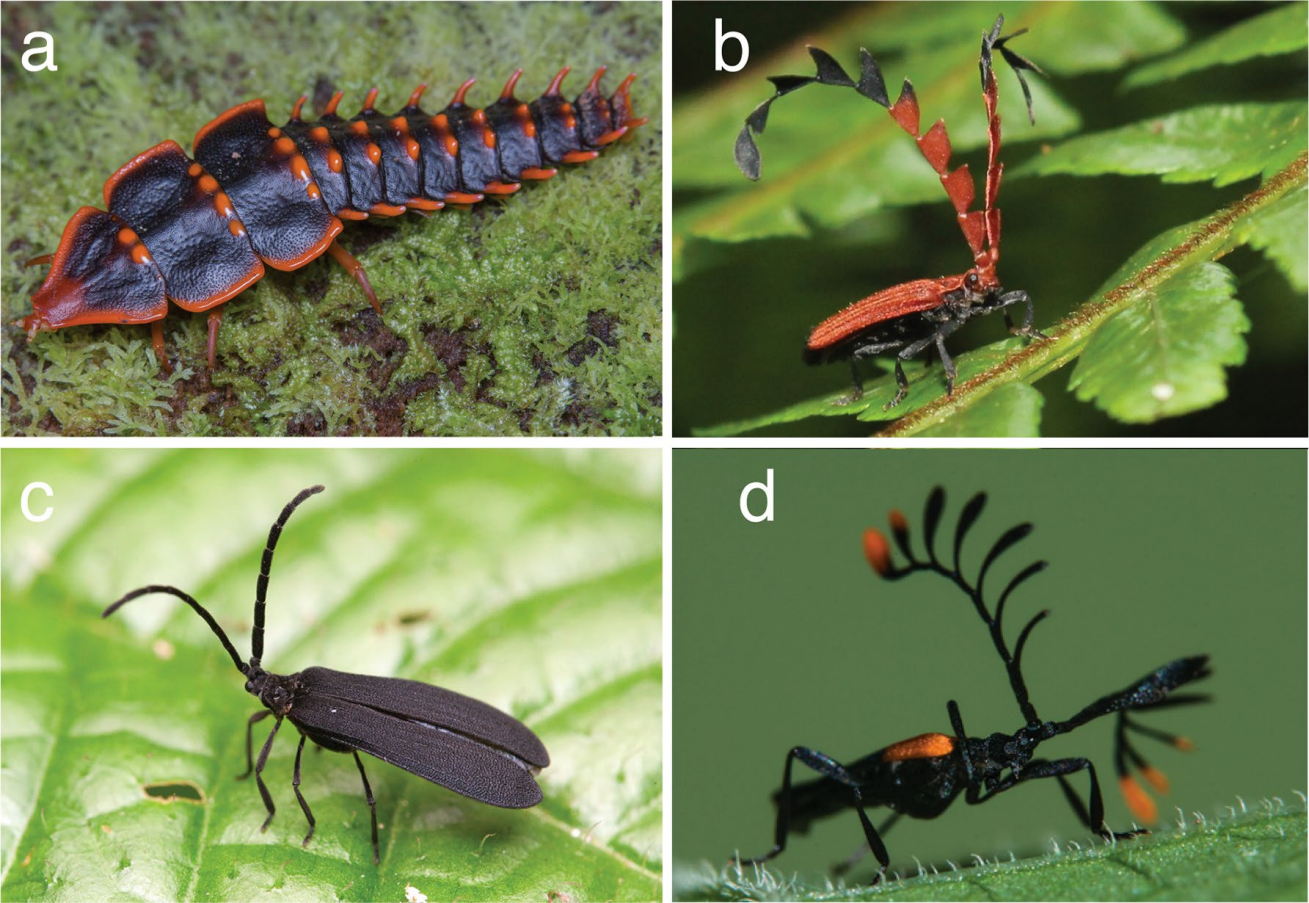
Lycidae known or suspected to be affected by the paedomorphic syndrome. (**A**) *Platerodrilus* sp. (image: Marcus F.C. Ng). (**B**) *Atelius expansicornis* Walker, 1869, Sinharaja Research Station, Ratnapura, Sri Lanka (image: Amila Sumanapala). (**C**) *Lyropaeus* sp. (image: Marcus F.C. Ng). (**D**) *Leptolycus* (*Baholycus*) *flavoapicalis* Bocak, 2001, Los Montones, Villa Altagracia, Dominican Republic (image: Carlos de Soto Molinari).

In net-winged beetles, a recent study indicated that paedomorphosis evolved at least four times independently in the family^15^, and most net-winged beetle lineages affected by the syndrome are found in the Old World, such as the Lyropaeinae, including the famous ‘trilobite larvae’ of *Platerodrilus* spp. (Fig. 1A,C) (Indomalayan region^8^), the Ateliinae (Fig. 1B) (Indomalayan and Australasian regions^16^), and Dexorinae (Afrotropical region^17^). In the New World, paedomorphic lineages are found only within the subfamily Lycinae, in numerous genera in the endemic Neotropical tribes Leptolycini (Fig. 1D) and Calopterini^18–25^.

One of the attributes that has been hypothesized for paedomorphic lineages is a tendency for rapid diversification and higher speciation rates^26–28^. In other animal groups, higher speciation rates have been correlated with higher morphological plasticity^29,30^. Fossils of the paedomorphic lineages could provide valuable palaeobiological data to investigate evolution of paedomorphosis and, in particular, to test the higher speciation rates hypotheses. In the Elateroidea fossil record, several taxa affected by the paedomorphic syndrome were found in various families^31–33^. In the described fossil record of Lycidae, currently comprised by 12 known species^20,34–37^, only three species were assigned to lineages that are known to have individuals affected by the paedomorphic syndrome, *i*.*e*., *Electropteron avus* Kazantsev, 2013 and *Cessator brodzinskyi* Ferreira and Ivie, 2017 (both Lycinae, Leptolycini) from Dominican amber^20,35,38^ and *Burmolycus compactus* Bocak et al*.*, 2019 (Dexorinae, Burmolycini) from Burmese amber^39^. Such records indicate that paedomorphosis has been occurring in this family at least since ca. 100 Mya. Our recent survey of collections of Dominican amber specimens revealed four undescribed specimens of Lycidae, including the first lycid larva that could be identified to generic level. A study of this material and comparison with previously described taxa allowed the placement of these undescribed taxa in the extant paedomorphic Greater Antillean genus *Cessator* Kazantsev, 2009 (Leptolycini).

Since *Cessator* is a well established paedomorphic lineage^38,40^, it can be predicted that in this poorly dispersing beetles affected by the paedomorphic syndrome, several million years old fossils would be morphologically rather distinct from extant congeners due to the increased speciation rate^41^. However, so far this does not seem to be the case found in the paedomorphic net-winged beetles from Dominican Amber^35,38,42^. So far, known Dominican amber lycid fossils challenge the hypothesis that lineages with presumed higher speciation and diversification rates should be correlated with an increased morphological plasticity over time, and the discovery of additional specimens is particularly intriguing. With all those controversies in mind, we not only provide descriptions of these newly found fossils but also propose potential explanations of the observed conserved and little changed morphology in paedomorphic lycids in the Greater Antilles from the time of the Dominican amber formation till present.

## Systematic paleontology

Order Coleoptera Linnaeus, 1758.

Family Lycidae Laporte, 1838.

Subfamily Lycinae Laporte, 1838.

Tribe Leptolycini Leng and Mutchler, 1922.

Genus *Cessator* Kazantsev, 2009.

(Figs. 2, 3, 4).

**Figure 2:**
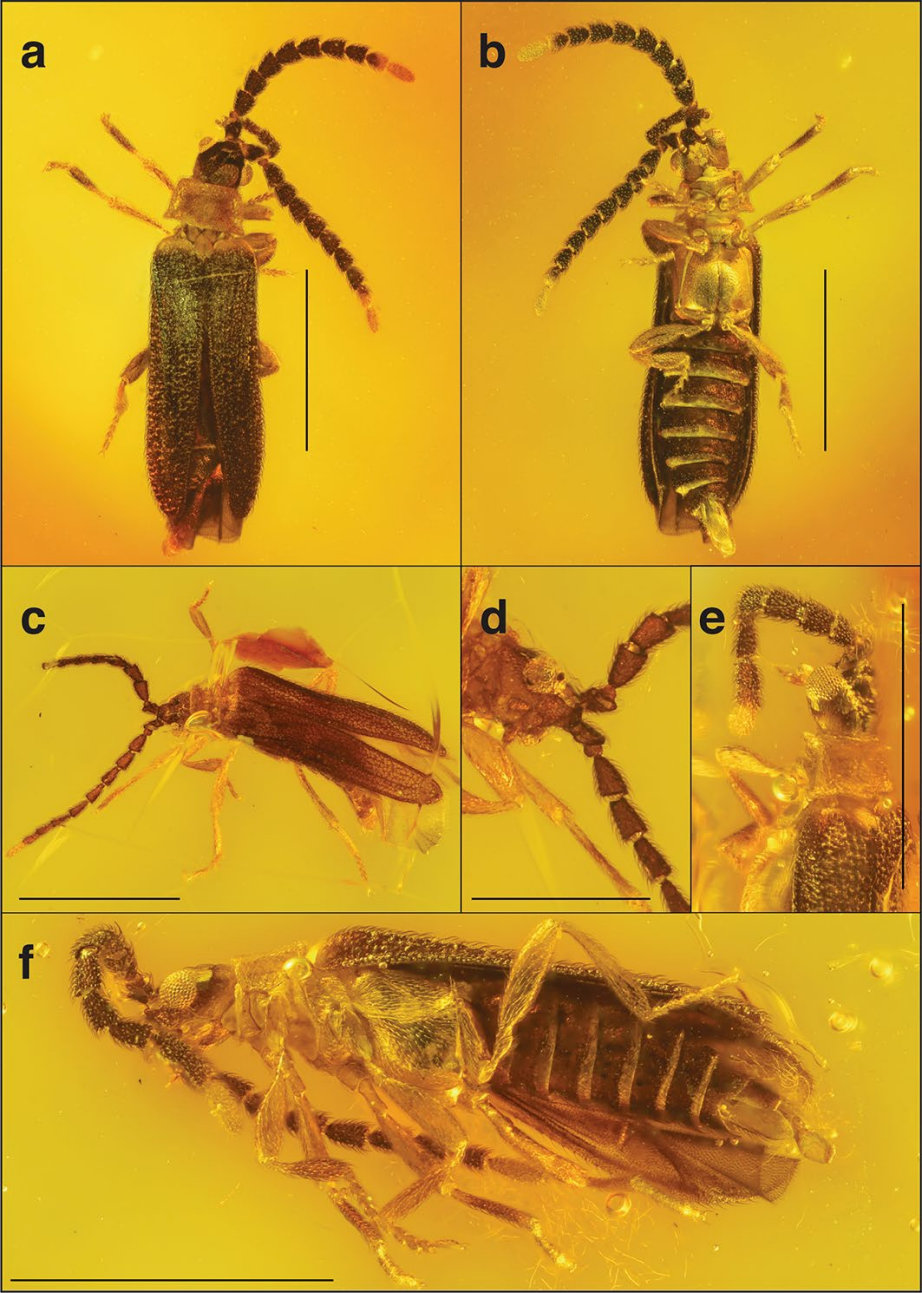
(**A**, **B**) Holotype of *Cessator anachronicus* sp. nov. (AMNH no. DR-10-648). (**A**) Habitus, dorsal view. (**B**) Habitus, ventral view. (**C**–**F**) Paratypes of *C*. *anachronicus* sp. nov. Paratype (AMNH no. DR-10-647) figures (**C**, **D**). (**C**) Habitus, dorsolateral view. (**D**) Ventral view of head and base of antennae. Paratype (AMNH no. DR-10-816) figures (**E**, **F**). (**E**) Lateral view of anterior part of body. (**F**) Habitus, ventrolateral view. Scale bars 1 mm, except Fig. 1D, which is 0.5 mm.

**Figure 3:**
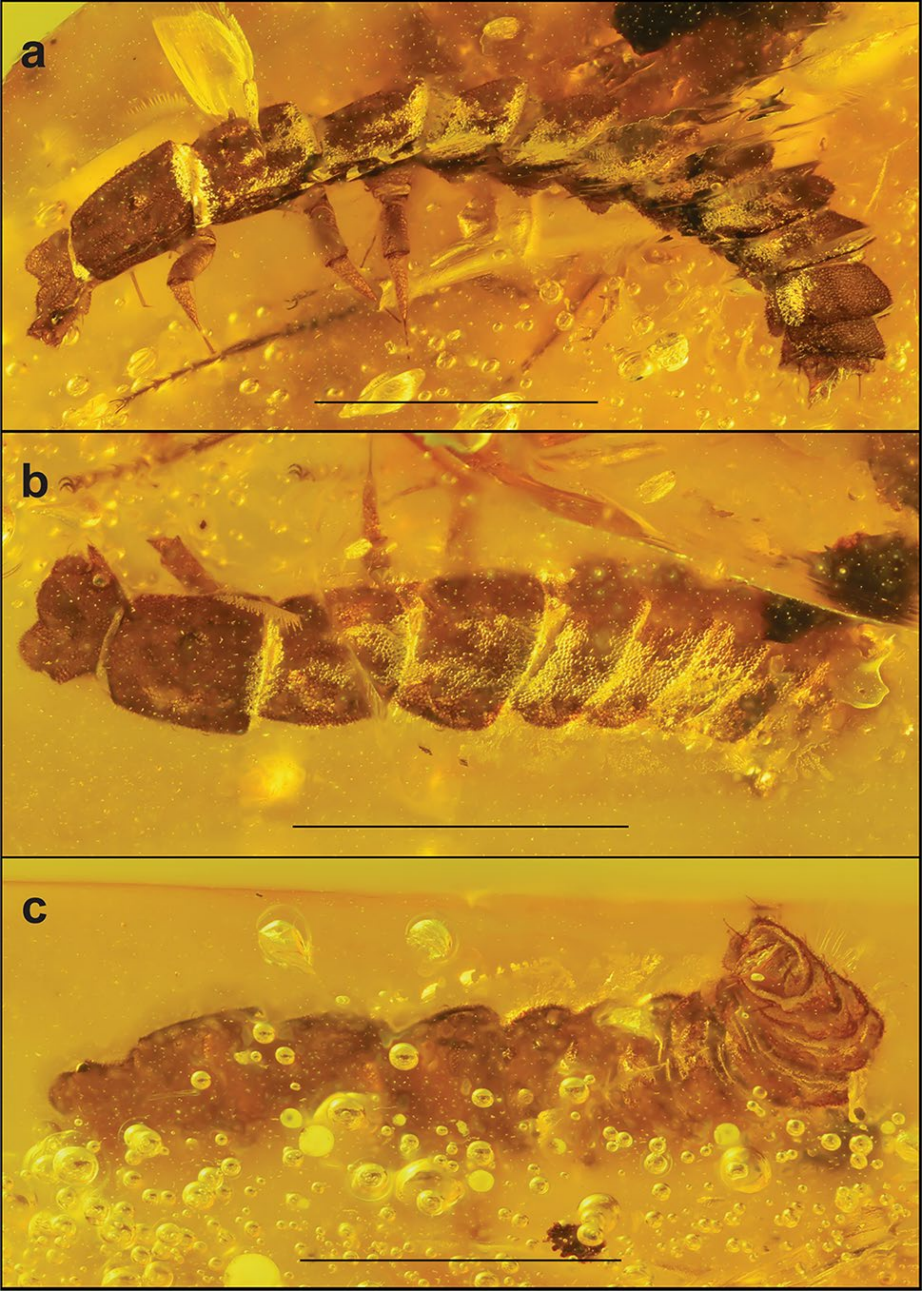
First fossil of a *Cessator* larva, habitus. (**A**) Dorsal view and detail of head. (**B**) Lateral view and detail of head and pronotum. (**C**) Ventral view and detail of the last abdominal segments.

**Figure 4:**
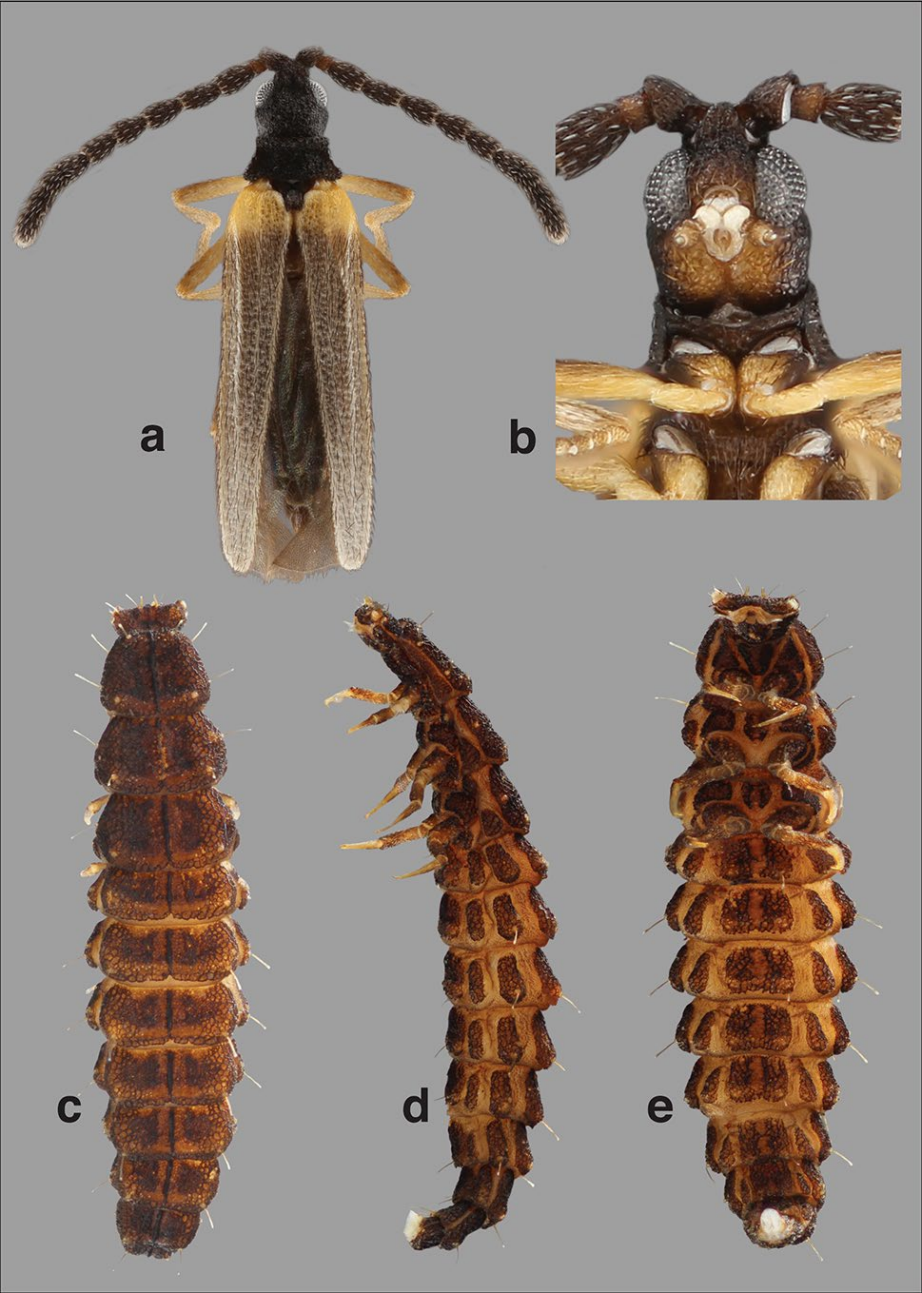
*Cessator luquillonis* Kazantsev, 2009 adult and larva from Puerto Rico. Male adult. (**A**) Habitus, dorsal view. (**B**) Head ventral view. Larva. (**C**) Dorsal view. (**D**) Lateral view. (**E**) Ventral view.

### ***Remarks***

*Cessator* is a West Indian genus of Lycidae currently known by one extant and two extinct species, including the one described here. The extant species, *Cessator luquillonis* Kazantsev, 2009, is known from the eastern part of Puerto Rico^40^, while the two fossils are from Dominican amber deposits. The diagnostic characters of the genus were given in Kazantsev^40^ and Ferreira and Ivie^38^ and will be expanded by Ferreira and Ivie^43^ in their review of the Puerto Rican Leptolycini study, where many other species and the immature forms of the group are going to be reported.

## *Cessator anachronicus* Ferreira and Ivie, sp. nov.

(Fig. 2D–F).

### Examined material

Holotype: Male, AMBER: Oligo-Miocene; Dominican Republic; AMNH no. DR-10-648; Locality: Unknown (AMNH). Two Paratypes: males, same data as for the holotype, no. DR-10-816 and no. DR-10-647 (both AMNH). Specimens are well preserved amber beads that have been polished prior to this study.

### Etymology

Anachronism is defined by the Cambridge dictionary as a person, thing or idea that exists out of its time in history. The species name was given in allusion to its remarkable similarity with extant congenerics, even though being set apart by millions of years. The specific epithet comes from the word anachronous, with its Greek roots meaning ἀνά ana, 'against', and χρόνος, khronos, 'time', which in its adjectivized form changes to *anachronicus*.

### Differential diagnosis

*Cessator anachronicus* sp. nov. can be distinguished from all other known fossil Leptolycini by its color combination, having the thorax, proximal region of the humeri, and apical half of antennomere X and antennomere XI yellow (Fig. 2A,B,E,F) (vs. variable in other fossil Leptolycini, the pronotum and ventral view of thorax in *Cessator brodzinskyi* dark brown), the pedicel roughly 1/3 the length of scape and antennomere III (Fig. 2A–F) (vs. pedicel and antennomere III subequal in length in *Electropteron avus* and in the undescribed Leptolycini sp. in Wu^44^), the antennomeres III–IX tubular and uncompressed, with even margins (Fig. 2A–F) (vs. antennomeres III–IX strongly compressed and flattened, bearing crenulate margins in *E*. *avus* and Leptolycini sp. in Wu^44^), the distinctly punctate elytra bearing two parallel costae that are feebly fused apically (vs. smooth elytra and elytral costae distinctly fused apically in Leptolycini sp. in Wu^44^) and the ventrite VIII apically round (Fig. 2B,F) (vs. lanceolate in *E*. *avus*). *Cessator anachronicus* sp. nov. can be separated from *Cessator luquillonis* (the only currently described *Cessator* species) by the pale pronotum (Fig. 2A,C,E) (vs. black pronotum in *C*. *luquillonis*, Fig. 4A), pale tips of the antennae (Fig. 2) (vs. antennae completely black in *C*. *luquillonis*, Fig. 4A), and by the non-confluent gular sutures, that are connected by an additional plate in *C*. *anachronicus* (vs. confluent gular sutures of *C*. *luquillonis*, Fig. 4B).

### Description

#### Male

Possessing all generic characteristics described in Kazantsev ^40^ and Ferreira and Ivie^43^. **General coloration** Head, antennae, apical half of pro-tibiae and ventrites I–VII brown (Fig. 2A,C,D); apical half of antennomere X on some specimens (Fig. 2F) and antennomere XI (Fig. 2A,B); lateral margins of head, gular region, thorax, legs, humeral region and genital capsule (ventrite VIII and sternites 9 and 10) yellow (Fig. 2A–F). Body moderately setose, bearing short decumbent setae (Fig. 2C,F). **Head** Antennae densely setose, with presence of scaliform setae (Fig. 2B,F). Antennae: approximate at base, subserrate, tubular, uncompressed, with even margins (Fig. 2A,B,D–F); scape ca. 3× longer than pedicel, pyriform (Fig. 2A,C,D); antennomere III ca. 3× longer than pedicel, which is minute (Fig. 2A,C,D); antennomeres IV–X subequal to III, slightly tapering towards apex, antennomere XI elongate, apically round (Fig. 2B,F). Head as long as wide, eyes protuberant, coarsely granulate (Fig. 2A,B,D–F); maxillary palp 4-segmented, terminal segment acuminate (Fig. 2B); gular sutures not confluents, connected by presence of gular bridge between sutures (Fig. 2B,F). **Thorax** Pronotum transverse, margins moderately developed (Fig. 2A,C,E), posteriorly bearing weakly developed median fovea (Fig. 2A); area adjacent to disc strongly punctate (Fig. 2A,C). Prosternum V-shaped, posteriorly acuminate (Fig. 2A,F). Mesoventrite trapezoidal, directly connected to mesanepisternum without additional segments (Fig. 2B,F). Metaventrite convex, posterolateral angles moderately acute, metadiscrimen complete (Fig. 2B,F); metanepisternum in lateral view crescent shaped, basal half anteriorly round, tapering towards apex, which is acuminate (Fig. 2B,F). Scutellar shield with shallow notch in posterior median portion, nearly cordiform (Fig. 2A). **Elytra** dehiscent, weakly ligulate, 2-costate, with short bristle-like setae throughout (Fig. 2F); costae weakly developed, subparallel and apically fused (Fig. 2F,C,E). **Abdomen** ventrite VIII ovate, apically blunt, one third longer than tergite 9 and one third shorter than tergite 10 (Fig. 2B).

**Length (head + pronotum + elytra):** 2.07–2.08 mm. **Width (across humeri):** 0.43–0.53 mm.

## First discovery of a leptolycini fossil larva

### *Cessator* sp.

(Fig. 3A–C).

### Examined material

AMBER: Oligo-Miocene; Dominican Republic; AMNH no. DR-15–11; Locality: Unknown (AMNH).

### Diagnosis

The *Cessator* larva herein studied is the only known fossil larva of Leptolycini ever found. It can be readily identified as a *Cessator* by possessing the characteristics described in Ferreira and Ivie^43^: the specimen has a small head, which is ca. half the length of the pronotum (Fig. 3A,B), the presence of paired projections in the anterior margin of the head (Fig. 3A), and the presence of long erect setae arising from the tergites and pleurites (Fig. 3C). The only generic characteristic not visible in the specimen studied is the presence of a dorsal and ventral median longitudinal line, however, this could simply be explained as an artifact of the fossilization process.

**Length:** 2.9 mm.

## Discussion

Fossil Lycidae are known from the Cretaceous and Tertiary deposits from various parts of the world^36^. The only known impression fossil is a poorly preserved specimen from the Eocene Florissant beds in North America^34^. However, its identity is too uncertain to be considered in our discussion. The remaining 11 species have been described from amber specimens: two species are known from Cretaceous Burmese amber (ca. 99 Mya), five from Eocene Baltic amber (ca. 30–50 Mya), three (including *C. anachronicus* herein described) from Miocene Dominican amber (as old as 30 Mya, see “Discussion” further in the text), and one from Miocene Mexican amber (ca. 23 Mya) (see detailed lists of amber Lycidae fossils in Kazantsev^35,42^, Bocak et al.^37^ and Li et al.^45^). Of these fossils, only the Leptolycini (Dominican amber) and Burmolycini (Burmese amber) are thought to be paedomorphic, either because specimens belong to recent lineages that are known to be paedomorphic (Leptolycini) or their morphology conforms with groups suspected to be paedomorphic (Burmolycini) (see examples in Ferreira et al.^23^ and Ferreira and Silveira^24^).

Among the described species of amber fossils of Lycidae, only four were assigned to their own new genera (*Cretolycus* Tihelka, Huang and Cai, 2019, *Burmolycus* Bocak, Li and Ellenberger, 2019 and *Murcybolus* Li, Tihelka, Huang and Cai, 2021 from the Cretaceous, and *Protolycus* Kazantsev, 2019 from Eocene), whilst all other species were described in genera for which one or more extant species are known. On the other hand, *Cessator anachronicus,* as well as the first reported fossil lycid larva herein described, can be readily assigned to an extant genus. The newly studied specimens have all the generic diagnostic characters of *Cessator* (Leptolycini) and are so similar to their extant congenerics that if individuals were not encased in amber, it would be impossible to determine that this species is extinct.

The remarkable similarity of *C*. *anachronicus* and also *C. brodzinskyi* with recent congenerics is not exclusive to this genus and this pattern can be found in various other known beetles from Dominican amber. In fact, the taxonomic placement of Dominican amber inclusions in extant genera of plants and insects is a common aspect of this fossil deposit (see references further in the discussion). The other known paedomorphic Dominican amber Leptolycini—*E*. *avus* and an undescribed species of Leptolycini from Wu^44^—also have the same conspicuous similarity with their respective congenerics, possessing all the modern features present in extant species. Outside of the Leptolycini, only a few morphological differences can be seen in *Plateros jardinesi* Kazantsev, 2020 from the Early Miocene (23–15 Mya) Mexican Chiapas amber and its recent congeners, and also between the majority of lycid species from Baltic amber (ca. 30–50 Mya) and their recent congeners, suggesting morphological stasis in these groups too.

These observations conflict with the seemingly established notion that beetle lineages affected by the paedomorphic syndrome are hypothesized to speciate faster and diversify more rapidly than non-paedomorphic groups^26,28–30,41^ due to their low dispersal capacity because of morphological modifications attributed to the paedomorphic syndrome, including flightlesness in females^6^. Indeed, poorly dispersing beetle lineages have a greater tendency to diversify^41^. The reduced mobility, interrupted gene flow and consequently limited geographical range has been reported to increase allopatric speciation and endemism rates in paedomorphic lycids^8,28^. Studies on other animals have also associated higher speciation and diversification rates with increased morphological modifications and phenotypical diversifications^29,30,46,47^. It can be therefore anticipated that lineages affected by the paedomorphic syndrome are expected to present a more diverse and divergent morphology. However, the examples found in *Cessator* and other fossil Leptolycini seem to contradict these assumptions, since very little morphological variation and divergence was found in the reported groups.

How can we explain these controversial observations, at least for the Greater Antilles lycid genera from the Dominican amber? One possible explanation is the relatively young age within the range of uncertainty associated with the age estimates of the Dominican amber. A younger age of the fossil within an uncertainty interval simply reduces time available for the lineages to diversify and develop their morphological diversity. In the early phase of speciation, lineages may differ only genetically but remain morphologically similar^28^. This notion may be relevant in support for the minimal age estimate of the Dominican amber in the controversy that places its age somewhere between 13 and 30 Mya^31,48–55^, with even younger ages proposed by Braga et al.^56^. Many other Dominican amber insects, including beetles from various families, have also been described in extant genera and they also displayed little morphological variation from their modern relatives (see review in Hörnschemeyer et al.^57^, and further examples in Poinar and Brown^58^, Tarasov et al.^59^, Keller and Skelley^60^, and Fanti and Pankowski^31^).

Another explanation, or rather additional factor enforcing the first explanation, is a presumed microhabitat stability of Greater Antilles tropical forest leaf litter from Chattian in Oligocene (i.e., the oldest time estimate for Dominican amber) until present. Previous studies^28,57,61,62^ suggested that microhabitat stability can be one of the factors that promote evolutionary stasis in beetles and other arthropods. The microhabitat conditions in the Greater Antilles, in which the Leptolycini can be found, with male adults flying in lower vegetation and larvae and females being found in leaf litter^40,63^, presumably persist since before the time of Dominican amber production. Dominican amber fossil plant records indicate stable composition of plant communities in the West Indies since the Miocene^64,65^, presumably harboring a similar leaf litter microhabitat structure where modern Leptolycini have been found.

While the interplay between the paedomorphosis and long-term persistence of microhabitats remains largely unknown and poorly discussed, the role of the presumed unchanged paleoecology of microhabitat could be more relevant than originally thought. The example of the Greater Antilles *Cessator* and the other known Leptolycini fossils may also indicate that groups affected by the paedomorphic syndrome, in such stable conditions, will not necessarily have a more divergent morphology linked to their presumed higher speciation and diversification rates. While there is some controversy as to whether or not higher diversification and speciation rates will necessary lead to morphological diversification (see discussions in Adams et al.^66^ and Beltrán et al.^30^), there are no studies similar to those cited above focused on relatively young group of paedomorphic insects, such as the Leptolycini.

It is important to stress that the scope of studies suggesting that paedomorphic Lycidae lineages do speciate rapidly has been limited, only focusing on groups that are entirely affected by paedomorphic processes, i.e., all ingroup taxa included in those studies were completely comprised of paedomorphic species^8,28^. In such studies, it is presumed that the paedomorphic syndrome evolved at the early stage of the evolutionary diversification in those groups, and all subsequent descendants (= terminals) simply retained the symplesiomorphic features associated with the syndrome. There are no studies on diversification and morphological variation for heterogenous paedomorphic groups (i.e., groups for which the paedomorphic syndrome has evolved independently more than once within the same lineage), such as presumably several South American paedomorphic Lycidae^18–25,67^. Further correlations between higher speciation and diversification rates and morphological diversity are still very limited in invertebrates, especially in beetles, and more in-depth and large-scale studies on these topics are yet to be proposed before broader extrapolations can be confidently made.

## Conclusion

The study of fossils is important not only because of the intrinsic improvement they generate on the taxonomic knowledge of a group, but also because they very often lead to clues to the evolutionary processes shaping the diversity of organisms. The description of *Cessator anachronicus* and of the first *Cessator* larva from Dominican amber represent further advances on the taxonomic knowledge of the poorly studied Leptolycini and in Lycidae overall, by providing further dating points that can be used to calibrate the phylogeny of the family. This study also provides further historical documentation of paedomorphosis in a Lycidae lineage from the West Indies, as well as evidence of morphological stasis in a group for which rapid acquisition of high phenotypic and morphological diversity was anticipated. Possible explanations for the observed morphological stasis in the Leptolycini involve the shorter period of time (ca. 15 myr) which elapsed from the time of Dominican amber deposition to present, i.e., not enough time to develop morphological diversity, and, more importantly, stability of the leaf-litter and forest microhabitats for tens of million of years in the West Indies. Even though the paedomorphic syndrome and microhabitat stability are expected to be antagonistic evolutionary drivers given some already published evidence, in *Cessator*, their interaction resulted in a case of morphological stasis, in which extant and extinct species are nearly identical. While this study does not provide any definitive answer for the questions herein raised, we hope it will draw attention to the interplay of these evolutionary drivers and their effects in beetles' morphology evolution.

## Material and methods

All four studied amber specimens are deposited in the Amber Collections of the American Museum of Natural History, New York, NY, USA (AMNH) and were sent in a loan for this study by Dr. David Grimaldi (curator). The exact locality of the specimens is unknown, and the only information provided regarding their age is that the samples are from the Oligocene–Miocene horizon.

The specimens were studied under a LeicaWild M3C stereoscopic microscope with magnification up to 40× and identified using available literature^20,21,35,38,43,68^ and by comparison of material with Leptolycini specimens assembled in the Montana Entomology Collection, Bozeman, MT, USA (MTEC). Adult morphological terminology follows Kazantsev^69^ and Lawrence et al.^70^, and immature terminology follows Ferreira and Costa^71^.

For photographs, specimens were completely submerged in olive oil. Photos were taken using a Canon 6D DSLR using the lens MP-E 65 mm and a Stackshot—automated macro rail for focus stacking. Images were processed with Zerene Stacker software version 1.04. Enhancements to digital images were made in Adobe Photoshop CC 2021 and final plates prepared using Adobe Illustrator CC 2021.

### Nomenclatural acts

This published work and the nomenclatural acts it contains have been registered in ZooBank. The LSID for this publication is urn:lsid:zoobank.org:pub:3E7ECBF4-8DFC-4F38-A54C-92E094D784D0.
